# Expansion of VariZIG distribution in the United States

**Published:** 2014-11-21

In July 2013, CDC published updated recommendations for the use of VariZIG for postexposure prophylaxis of varicella for persons at high risk for severe disease who lack evidence of immunity to varicella and for whom varicella vaccine is contraindicated (1). At the time of the recommendation, VariZIG was available from only one U.S. distributor (FFF Enterprises; Temecula, California; telephone, 800-843-7477; online at http://www.fffenterprises.com). Now, VariZIG is also available from a second distributor (ASD Healthcare; Frisco, Texas; telephone, 800-746-6273; online at http://www.asdhealthcare.com).
